# Static and Dynamic Analysis of a Bistable Frequency Up-Converter Piezoelectric Energy Harvester

**DOI:** 10.3390/mi14020261

**Published:** 2023-01-19

**Authors:** Mohammad Atmeh, Alwathiqbellah Ibrahim, Abdallah Ramini

**Affiliations:** 1 Department of Mechanical Engineering, The University of Texas at Tyler, 3900 University Blvd., Tyler, TX 75799, USA; 2 Shock and Vibration Lab-IBM Corporation, 2455 South RD, Poughkeepsie, NY 12601, USA

**Keywords:** piezoelectric, energy harvesting, up-conversion, bistable, magnet

## Abstract

Using energy harvesting to convert ambient vibrations efficiently to electrical energy has become a worthy concept in recent years. Nevertheless, the low frequencies of the ambient vibrations cannot be effectively converted to power using traditional harvesters. Therefore, a frequency up-conversion harvester is presented to convert the low-frequency vibrations to high-frequency vibrations utilizing magnetic coupling. The presented harvester consists of a low-frequency beam (LFB) and a high-frequency beam (HFB) with identical tip magnets facing each other at the same polarity. The HFB, fully covered by a piezoelectric strip, is utilized for voltage generation. The dynamic behavior of the system and the corresponding generated voltage signal has been investigated by modeling the system as a two-degrees-of-freedom (2DOF) lumped-parameter model. A threshold distance of 15 mm that divides the system into a monostable regime with a weak magnetic coupling and a bistable regime with a strong magnetic coupling was revealed in the static analysis of the system. Hardening and softening behaviors were reported at the low frequency range for the mono and bistable regimes, respectively. In addition, a combined nonlinear hardening and softening behavior was captured for low frequencies at the threshold distance. Furthermore, a 100% increment was achieved in the generated voltage at the threshold compared to the monostable regime, and the maximum generated voltage was found to be in the bistable regime. The simulated results were validated experimentally. Moreover, the effect of the external resistance was investigated, and a 2 MΩ resistance was found to be optimal for maximizing the generated power. It was found that frequency up-converting based on magnetic nonlinearity can effectively scavenge energy from low-frequency external vibrations.

## 1. Introduction

The continuous development in technology has recently led to improved power generation resources by transferring them from conventional to non-conventional. Conventional power resources, such as batteries, are limited due to their short lifespans and the ability to store power, so they require charging overtime. Therefore, harvesting energy from the ambient is an attractive concept that could remove the need for batteries and their limitations [1]. Mechanical vibrations are considered one of the most wasted energies that are abundant around us in the environment. They are present during our daily activities, such as driving and walking [2,3,4]. Consequently, research is constantly attempting to convert those mechanical vibrations to electrical energy to power wireless sensors and electronics that work in the micro-to-milliwatts range [5].

Nature is rich with mechanical vibrations that are abundant at low-frequency ranges [6]. Thus, benefits from ambient energy by harvesting those vibrations will provide a non-conventional power resource [7]. Different mechanisms are used to convert mechanical vibrations into electrical power. Electromagnetic [8], triboelectric [9,10], and piezoelectric [11] mechanisms are the most common. Among all these mechanisms, piezoelectric energy harvesters have the ability to work at a low power level and resist environmental conditions; therefore, they have been utilized in several environmental applications, such as being implanted inside the human body [12,13], civil infrastructure [14,15], and aerospace systems [16,17,18,19,20]. However, some drawbacks include the high resonance frequency which is away from the ambient ranges [2,21] and the narrow bandwidth [22]. Therefore, several techniques have been utilized to overcome these drawbacks, such as the nonlinearity contribution [23,24,25,26,27,28,29,30], circuit management [31], the double pendulum system [32], frequency-tunable oscillators [33,34,35], and frequency up-conversion [36]. Nonlinearity has contributed to expanding the bandwidth of the output power by influencing the mechanical [37,38], impact [39], and magnetic [30,40,41] effects.

To increase the efficiency of harvesting energy from low-frequency vibrations, the frequency up-converter has been investigated lately [36,42,43,44,45,46,47,48,49,50]. Basically, the approach of the frequency up-converter is that the low-frequency sources induce high-frequency oscillations [51]. This inducing can be obtained either by the impact [52] or by plucking [53]. An impact between low-frequency and high-frequency beams will change the ambient vibrations into high-frequency vibrations [42,54]. This would improve the low-frequency vibration harvesters, filling the gap between the low-frequency excitation and high-frequency response [55,56]. In addition, to increase the reliability and eliminate the issues of mechanical contact, magnetic coupling has been investigated as a non-mechanical contact method [36,45,57,58]. The low-frequency motions of animals were up-converted through a self-powered magnetoacoustic into high-frequency acoustic signals [59]. On the other hand, an electromagnetic frequency up-converter was used with magnets and coils on top of a resonator beam to generate power [58].

The magnetic nonlinear behaviors of softening and hardening can be achieved by controlling the separation distance between two magnets facing each other at the same polarity [30]. Broadening the bandwidth has been investigated by using microscale [60,61,62] and macroscale [21] monostable energy harvesters. Additionally, bistability was used to increase the frequency bandwidth and the magnitude of the output power [22,63,64,65,66,67,68,69]. Experimental results show that the maximum power is produced at the transition region between the monostable and the bistable regime [30,70,71]. Additionally, external load resistance was optimized in a magnetically coupled, two-degrees-of-freedom, bistable energy harvester to maximize the output power [72]. In addition, the effect of spring stiffness at low excitation frequencies under Gaussian white noise excitation causes a significant improvement in the harvesting efficiency of bistable energy harvesters [73].

In this study, we propose a frequency up-conversion method to harvest low-frequency ambient vibrations utilizing piezoelectric material for voltage generation. The frequency up-converter couples two cantilever beams mechanically using two identical magnets to convert low-frequency vibrations to high-frequency oscillations. The two cantilever beams have identical tip magnets facing each other at the same polarity, which induces magnetic coupling between both beams’ oscillations. The system of beams was modeled as a two-degree-of-freedom (2DOF) lumped-parameter model to investigate the dynamic behavior and generated voltage signal.

The remainder of this article begins with presenting the configuration of the device and the working mechanism of the frequency-up-converter energy harvester. The static response of each beam, the frequency variation and separation distance relation, and the voltage generation at different separation distance values are explored. Additionally, the simulation results from the theoretical model are validated experimentally.

## 2. Device Configuration and Principle of Operation

**Figure 1:**
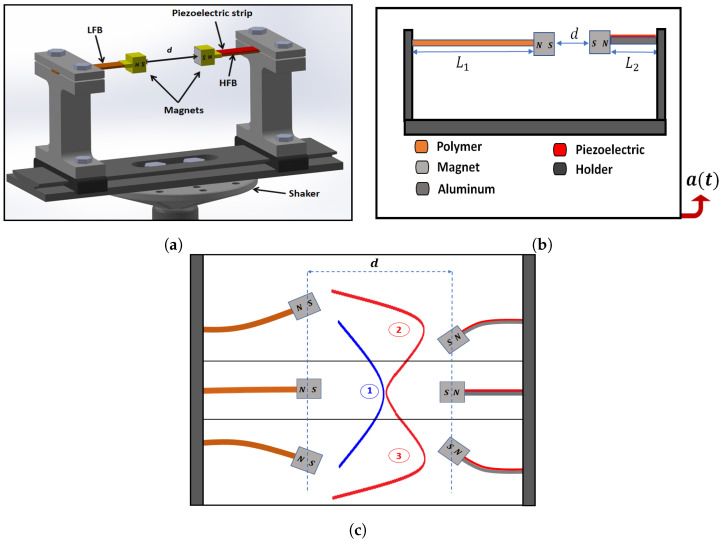
(**a**) A 3D model of the energy harvester system. (**b**) A 2D schematic of the energy harvester system. (**c**) The mono and bistable principles of operation under repulsive magnetic force.

A 3D model and a 2D schematic for the system are shown in Figure 1a and Figure 1b, respectively. The system consists of two cantilever beams with identical masses attached to the beams’ tips, as shown in Figure 1a. In Figure 1b, the beam is unimorph and fully covered by a piezoelectric strip to generate voltage, and it is made of aluminum for higher natural frequency and is called the high-frequency beam (HFB). On the left-hand side, the beam is made of a polymer material for lower natural frequencies and it is called the low-frequency beam (LFB). The tip masses are two identical magnets facing each other at the same polarity to utilize the generated repulsive force to create a nonlinear mechanical coupling that will transfer the energy between both beams to create a frequency-up-converter energy harvester. L1 and L2 are the lengths of the LFB and HFB, respectively. The beams are separated horizontally by a distance *d*, and the piezoelectric strip is connected to an external resistor *R*. The beams are attached to a holder, and the whole setup is installed on an electrodynamic shaker, as shown in Figure 1a. The system was subjected to a base excitation level (a(t)) applied by the electrodynamic shaker.

When the separation distance, *d*, decreases, the repulsive magnetic force between the tip magnets will increase and induce a strong nonlinearity in the system that will transfer the potential energy of the resonator from a monostable phase (the resonator oscillates around a single equilibrium point) to a bistable phase (the resonator oscillates around two equilibrium points). When the separation distance increases, the magnetic force will be weak, and the system will oscillate in a monostable phase where each beam will oscillate around its horizontal axis around a single-well stable equilibrium point, as shown in Figure 1c, case 1. On the other hand, decreasing the separation distance will strengthen the magnetic force so each beam will oscillate around two stable points, either above or below the horizontal axis, as in cases 2 and 3 in Figure 1c, so two potential wells will be developed for each beam.

## 3. Theoretical Model

**Figure 2:**
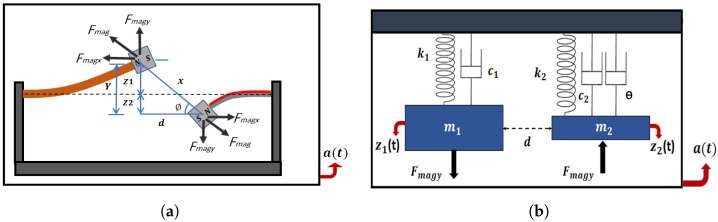
(**a**) Magnetic interactions between LFB and HFB. (**b**) Two-degrees-of-freedom spring-mass-damper systems for the LFB and HFB.

The shaker excites the frequency up-converter that consists of two cantilever beams with identical tip magnets facing each other at the same polarity. Both beams will oscillate, and the energy will be transferred from the low-frequency range to the high-frequency range due to the magnetic coupling between the tip magnets. This oscillation will develop the required stresses and strains to bend the beams to generate a voltage from the piezoelectric strip attached to the HFB, as shown in Figure 2a. The tip magnets will affect each other by a repulsive magnetic force with the same magnitude because they are identical, and the produced forces can be analyzed as vertical and horizontal components; see Figure 2a. Based on its geometry, the frequency up-converter is modeled as a two-degrees-of-freedom (2DOF) lumped-parameter model subjected to harmonic base excitation, as shown in Figure 2b.

From Figure 2b, the LFB and HFB are modeled as two-degrees-of-freedom (2DOF) spring-mass-damper systems. Accordingly, the governing equations that describe the system, including the coupling between the HFB and the piezoelectric strip, are as follows [72]:

(1) $$\begin{array}{c} \begin{array}{cc} \; & m_{1}\ddot{z_{1}}\left(t\right)+c_{1}\dot{z_{1}}\left(t\right)+k_{1}z_{1}\left(t\right)+F_{magy}=m_{1}a\left(t\right)\\ \; & m_{2}\ddot{z_{2}}\left(t\right)+c_{2}\dot{z_{2}}\left(t\right)+k_{2}z_{2}\left(t\right)+\theta v\left(t\right)-F_{magy}=m_{2}a\left(t\right)\\ \; & \dot{v}\left(t\right)-\chi \dot{z_{2}}\left(t\right)+\lambda v\left(t\right)=0 \end{array} \end{array}$$

where a(t) is the harmonic base excitation (a(t)=Acos(Ωt)), Ω is the excitation frequency, and *A* is the amplitude. $z_{1}\left(t\right)$ and $z_{2}\left(t\right)$ are the displacements of the LFB and the HFB, respectively. Additionally, $m_{1}$ and $k_{1}$ are the equivalent mass and the stiffness of the LFB, respectively, and $m_{2}$ and $k_{2}$ are the equivalent mass and the stiffness of the HFB, respectively. The damping coefficients for the LFB and HFB are $c_{1}$ and $c_{2}$, respectively. Additionally, v(t) is the voltage generated from the piezoelectric layer, and $\lambda =1/RC_{p}$, such that *R* is the resistance and $C_{p}$ is the capacitance ($C_{p}=\left(\epsilon_{33}b_{p}L_{p}\right)/h_{p}$), where $\epsilon_{33}$ is the permittivity ($3250\times 8.854\times 10^{-12}$); $b_{p}$, $L_{p}$, and $h_{p}$ are the piezoelectric layer’s width, length, and thickness, respectively. Additionally, χ can be calculated as $\chi =\frac{\theta }{C_{p}}$, where θ is the coupling coefficient of the piezoelectric layer. Additionally, the fundamental natural frequencies of both beams can be calculated from the following formula:

(2) $$f_{i}=\frac{1}{2\pi }\sqrt{\frac{k_{i}}{m_{i}}}\;,\;i=1,2.$$

where $m_{i}$ and $k_{i}$ are the equivalent mass and the stiffness of the beams, respectively. For the LFB, the stiffness can be calculated as ($k_{1}=3E_{1}I_{1}/L_{1}^{3}$) [74], and for the HFB, it can be calculated as ($k_{2}=3E_{p}I_{eq}/L_{2}^{3}$). The stiffness of the HFB represents the equivalent stiffness of the piezoelectric layer and the HFB beam. The HFB’s equivalent mass consists of the piezoelectric layer mass ($m_{p}$) and the effective mass of the cantilever beam ($m_{heff}=0.375m_{b}$) [74], where $m_{b}$ is the beam mass. $E_{p}$ represents the modulus of elasticity of the piezoelectric strip. $I_{eq}$ is the equivalent moment of inertia of the HFB and piezoelectric strip and can be calculated using Equation (3) [74].

(3) $$I_{eq}=\frac{W_{p}t_{p}^{3}}{12}+{\left(t_{p}W_{p}\left(t_{h}+\frac{t_{p}}{2}\right)-Y_{n}\right)}^{2}+\frac{nW_{p}t_{h}^{3}}{12}+t_{h}nW_{p}{\left(\frac{t_{h}}{2}-Y_{n}\right)}^{2}$$

where $Y_{n}$ is the location of the neutral axis of the HFB with the piezoelectric strip and can be calculated using Equation (4). Additionally, $t_{p}$ and $t_{h}$ are the thickness of the piezoelectric strip and the HFB, respectively. $W_{p}$ represents the width of the piezoelectric strip, and *n* is the ratio of the modulus of elasticity of the HFB to the modulus of elasticity of the piezoelectric strip ($n=E_{h}/E_{p}$).

(4) $$Y_{n}=\frac{\left(\frac{t_{p}}{2}+t_{h}\right)t_{p}+\left(\frac{t_{h}}{2}\right)nt_{h}}{\left(t_{p}+t_{h}n\right)}.$$

By evaluating the neutral axis location, $Y_{n}$, the electro-mechanical coupling factor, θ, can be calculated as:

(5) $$\theta =d_{31}\left(\frac{k_{2}b_{2}L_{2}\left(L_{2}+L_{p}\right)Y_{n}}{2I_{eq}}\right)$$

where $d_{31}$ is the piezo strain constant. Additionally, $b_{2}$ is the width of the HFB and $L_{p}$ is the length of the piezoelectric strip. The coupling magnetic force is a function of the separation distance (*d*) between the LFB and HFB beams, and it can create either a mono or bistable system as demonstrated in Figure 1c. This magnetic force can be calculated as [30]:

(6) $$F_{mag}=\frac{F_{R}}{X^{4}}$$

where *X* is the distance between the centers of the two magnets ($X=\sqrt{d^{2}+Y^{2}}$), and *Y* is the total vertical deflection between the two tip magnets given by $Y=z_{1}\left(t\right)+z_{2}\left(t\right)$. $F_{R}$ is a function of the moments for magnetic dipoles ($q_{1}$ and $q_{2}$) and the permeability of the free space ($\epsilon =4\pi \times 10^{-7}$ m kg/s${}^{2}$A${}^{2}$), and is given by $F_{R}=\left(3\epsilon q_{1}q_{2}\right)/2\pi$. According to Figure 2a, the total magnetic force can be analyzed using the angle ϕ into a horizontal component $F_{magx}$, which is assumed to be equivalent to the longitudinal stiffness of the beams, and a vertical component ($F_{magy}$) in the transverse direction, which is responsible for the beams transverse deflections and is given by the following equation:

(7) $$F_{magy}=\frac{F_{R}Y}{{\left(d^{2}+Y^{2}\right)}^{5/2}}$$

## 4. Experimental Setup

**Figure 3:**
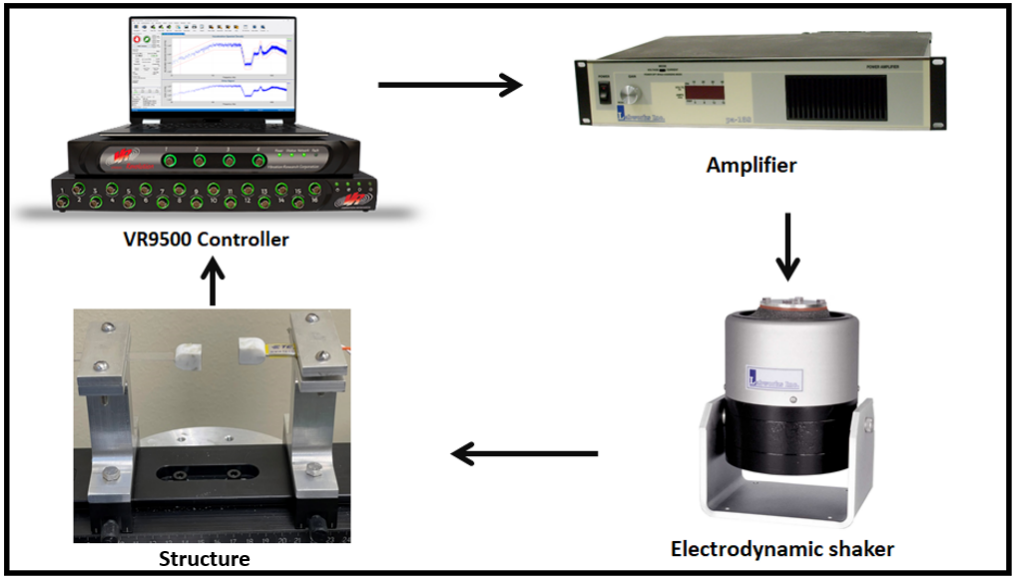
Experimental setup used to test the piezoelectric energy harvester frequency up-converter.

The experimental setup used for testing the harvester is shown in Figure 3. The setup consists of the VR9500 control unit, amplifier, electrodynamic shaker, and energy harvesting structure. The control unit controls the amplitude and frequency of the base excitation applied by the electrodynamic shaker. The amplifier receives the signal from the control unit and then it sends it to the shaker. Once the shaker receives the signal from the amplifier, it starts shaking and acting as a base excitation for the harvester structure. The frequency response curves of the LFB and HFB beams are measured by accelerometers attached to the tip magnet of each beam. The accelerometers are connected to the VR9500 control unit and measure the beams’ vibrations as a function of the excitation frequencies. Additionally, the voltage is generated by a piezoelectric strip attached to the HFB. When the beam oscillates, its deflection develops stress and strain on the piezoelectric strip, resulting in an alternative voltage signal being generated and recorded by the controller.

## 5. Results and Discussion

### 5.1. Static Analysis

**Figure 4:**
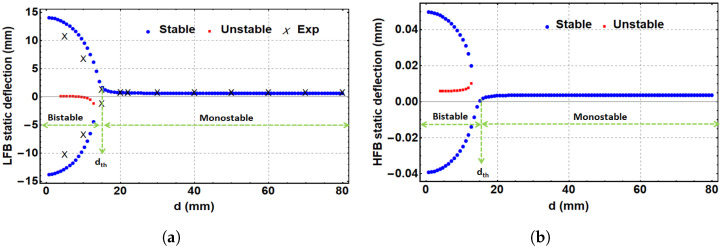
The static response of the LFB and HFB versus the separation distance. (**a**) Experimental and theoretical static response of the LFB, (**b**) theoretical response of the HFB. Threshold value, $d_{th}$, found to be 15 mm.

By setting all the time-derivatives to zero in Equation (1), the static response of both beams, the LFB and HFB, under the effect of the magnetic coupling, can be calculated as follows:

(8) $$\begin{array}{c} \begin{array}{cc} \; & k_{1}z_{1s}+F_{magys}=0\\ \; & k_{2}z_{2s}-F_{magys}=0 \end{array} \end{array}$$

where $z_{1s}$ and $z_{1s}$ represent the static deflections of the LFB and HFB, respectively. $F_{magys}$ is the static magnetic force in the vertical direction, and it is given by the following formula:

(9) $$F_{magys}=\frac{F_{R}Y_{s}}{{\left(Y_{s}^{2}+d^{2}\right)}^{5/2}}$$

where $Y_{s}$ is the static deflection of both beams between the magnets’ centers and is given by $Y_{s}=z_{1s}+z_{2s}$. Using the geometrical parameters listed in Table 1, the static solution for Equation (8) can be extracted. Figure 4a,b show the theoretical and experimental static response of the LFB and the theoretical static response of the HFB, respectively, with the variation in the horizontal distance between the two tip magnets. It is clearly shown that a critical threshold separation distance is $d_{th}=15$ mm. This threshold distance divides the system into two main parts, a monostable region where $d>d_{th}$ and a bistable region where $d<d_{th}$. In the monostable region, the static profiles of both beams show single stable branches, where both beams oscillate around a single equilibrium point in the middle. In the bistable range, each static profile shows two upper and lower stable branches and one unstable branch in the middle. Each beam will oscillate between the two stable equilibrium points in the bistable range. Figure 4a shows good agreement between the theoretical and experimental results of the LFB. The experimental measurements of the LFB were taken by measuring the separation distance (*d*) between the centers of both tip magnets using a ruler. Once the LFB starts showing a significant deflection by moving towards the threshold and bistable regions, the vertical deflection of the LFB gets measured between the original horizontal axis of the beam and the new location of the tip magnet center. The HFB is a stiff beam with minimal deflections that may need an advanced measurement system, such as one using laser technology, to be captured experimentally. However, the match between the experimental and simulated results for the LFB indicates our model’s ability to capture the correct static behavior for the HFB.

### 5.2. Dynamic Analysis

#### 5.2.1. Natural Frequencies

To solve for the natural frequencies of the system as a function of the separation distance, we assume the total deflection of the beams to be a function of the static and dynamic deflections as:

(10) $$\begin{array}{c} \begin{array}{cc} \; & z_{1}\left(t\right)=u_{1}+z_{1s}\\ \; & z_{2}\left(t\right)=u_{2}+z_{2s} \end{array} \end{array}$$

where $u_{1}$ and $u_{2}$ are the dynamic deflections for the LFB and HFB, respectively. Accordingly, the total vertical deflection will be:

(11) $$\begin{array}{c} Y=Y_{s}+Y_{u}. \end{array}$$

where $Y_{u}$ is dynamic deflection and given by $Y_{u}=u_{1}+u_{2}$. By substituting Equation (11) into Equation (9), the vertical magnetic force will be:

(12) $$\begin{array}{c} F_{magy}=\frac{F_{R}\left(Y_{s}+Y_{u}\right)}{{\left(d^{2}+{\left(Y_{s}+Y_{u}\right)}^{2}\right)}^{5/2}} \end{array}$$

Substituting Equations (2)–(12) into Equation (1) will result in the following governing equation:

(13) $$\begin{array}{c} \begin{array}{cc} \; & m_{1}\ddot{u_{1}}\left(t\right)+c_{1}\dot{u_{1}}\left(t\right)+k_{1}\left(u_{1}+z_{1s}\right)+F_{magy}=m_{1}a\left(t\right)\\ \; & m_{2}\ddot{u_{2}}\left(t\right)+c_{2}\dot{u_{2}}\left(t\right)+k_{2}\left(u_{2}+z_{2s}\right)+\theta v\left(t\right)-F_{magy}=m_{2}a\left(t\right)\\ \; & \dot{v}\left(t\right)-\chi \dot{u_{2}}\left(t\right)+\lambda v\left(t\right)=0 \end{array} \end{array}$$

To cancel the static effect according to the static Equation (Equation (10)), and to avoid the complications of the magnetic formula in getting the numerical solution, the magnetic force, $F_{magy}$, was expanded using Taylor’s series around zero dynamic deflection ($Y_{u}=0$) for up to nine terms. Accordingly, the magnetic force will be:

(14) $$\begin{array}{c} \begin{array}{cc} F_{magy} & =\frac{F_{R}Y_{s}}{{\left(d^{2}+Y_{s}^{2}\right)}^{5/2}}+\sum_{i=1}^{9}\alpha_{i}Y_{u}^{i}\left(t\right),\;\;\;i=1,2,\ldots 9\\ \; & =F_{magys}+F_{magyu} \end{array} \end{array}$$

where $\alpha_{i}$ are the coefficients of Taylor’s series expansion of the dynamic magnetic force, and they are listed in Appendix A. $F_{magys}$ and $F_{magyu}$ represent the static and dynamic magnetic force, respectively. When Equation (14) is substituted into Equation (13), the static terms will cancel each other since they are equal to zero, as shown in Equation (8); therefore, the governing equations of the system will be:

(15) $$\begin{array}{c} \begin{array}{cc} \; & m_{1}\ddot{u_{1}}\left(t\right)+c_{1}\dot{u_{1}}\left(t\right)+k_{1}u_{1}+F_{magyu}=m_{1}a\left(t\right)\\ \; & m_{2}\ddot{u_{2}}\left(t\right)+c_{2}\dot{u_{2}}\left(t\right)+k_{2}u_{2}+\theta v\left(t\right)-F_{magyu}=m_{2}a\left(t\right)\\ \; & \dot{v}\left(t\right)-\chi \dot{u_{2}}\left(t\right)+\lambda v\left(t\right)=0 \end{array} \end{array}$$

Now, by substituting the dynamic magnetic force of Equation (14) in Equation (15), and using $Y_{u}=u_{1}+u_{2}$, the nonlinear natural frequencies of the system of the LFB and HFB, respectively, can be calculated as follows:

(16) $$f_{1}=\frac{1}{2\pi }\sqrt{\frac{k_{1}+\alpha_{1}}{m_{1}}},\;f_{2}=\frac{1}{2\pi }\sqrt{\frac{k_{2}-\alpha_{1}}{m_{2}}}.$$

where the term $\alpha_{1}$ is the coefficient of the linear term after expansion of the magnetic force with Taylor’s series and is given by:

(17) $$\alpha_{1}=\frac{F_{R}\left(d^{2}-4Y_{s}^{2}\right)}{{\left(d^{2}+Y_{s}^{2}\right)}^{7/2}}$$

**Figure 5:**
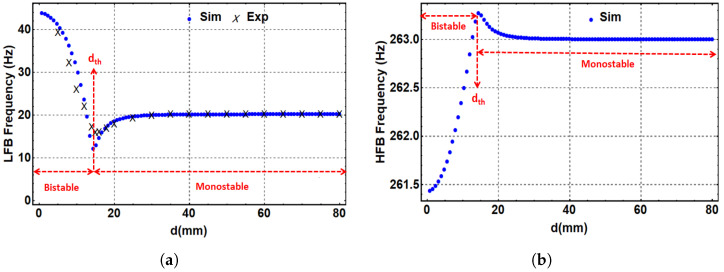
(**a**) The simulated and experimental variations in the LFB’s natural frequency with the separation distance. (**b**) The simulated variation in the HFB’s natural frequency with the separation distance.

Using Equation (16), the variation in the natural frequencies of the LFB and HFB with the separation distance between the two magnets is extracted and shown in Figure 5a,b, respectively. Additionally, the experimental variation in the natural frequencies with separation distance was recorded at a 0.1 g excitation level and reported for the LFB, as shown in Figure 5a. In contrast, the experimental natural frequency range of the HFB was hard to obtain due to the very small change in its natural frequency while changing the separation distance between the two magnets. Nevertheless, the LFB’s experimental and simulated results show good agreement. Both plots of the LFB and HFB show a threshold separation distance of 15 mm, which matches the same value extracted from the static results shown in Figure 4. Additionally, Figure 5 shows that at a large separation distance, the magnetic force becomes weak, and the natural frequencies match the linear values of both beams. Lowering the separation distance toward the threshold, the natural frequency of the LFB drops to reach a minimum value of 12 Hz, and the natural frequency of the HFB reaches its maximum value of 263.2 Hz. Decreasing the separation distance more to the bistable range will increase the natural frequency of the LFB to a maximum value of 43 Hz and drop the natural frequency of the HFB to a minimum value of 261.4 Hz. This change in the natural frequencies of the LFB and HFB is because of the contribution of the magnetic force term ($\alpha_{1}$) in Equation (17) that changes its sign according to the value of *d*, which results in lowering or increasing the beams’ natural frequencies. It is also noted that the natural frequency variation in the LFB is much more significant than that in the HFB due to the LFB’s low-stiffness material (polymer) compared to the HFB’s high-stiffness material (aluminum). It is obvious that the simulated data are not in perfect accordance with the experimental data in Figure 5a. Certainly, there are some discrepancies between the experimental and simulated data, which could be from the theoretical lumped-parameter model, which assumes the distributed mass of the beam to be lumped at one point. Moreover, the lack of accuracy in the magnetic force function used in the theoretical model could be another reason for the discrepancies between the theoretical and experimental results.

#### 5.2.2. Linear Analysis

**Figure 6:**
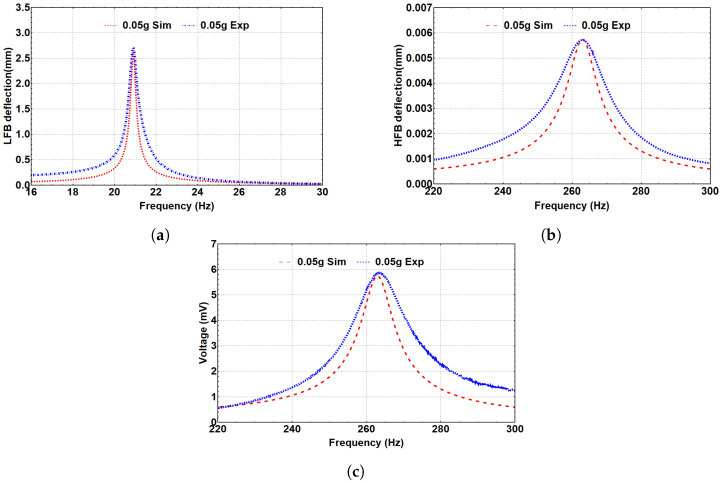
(**a**) Frequency response curve of the LFB. (**b**) Frequency response curve of the HFB. (**c**) Frequency–voltage curve of the HFB. The excitation level is 0.05 g.

The linear response of the system and the generated voltage can be investigated by eliminating the effect of the magnetic force ($F_{magy}=0$). Figure 6 summarizes the simulated and experimental frequency response curves of the LFB and HFB, and the frequency–voltage curve at a low excitation level of 0.05 g; all theoretical and experimental results are in good agreement. Figure 6a shows the LFB’s theoretical and experimental frequency response curves, where the natural frequency is 20.8 Hz with a maximum deflection of 2.75 mm. Similarly, Figure 6b shows the simulated and experimental frequency response curves for the HFB with a natural frequency of 263 Hz and 0.0055 mm maximum deflection. Additionally, Figure 6c represents the frequency–voltage curve generated from the piezoelectric layer, where the voltage peaks approximately at 263 Hz with 5.9 mV as a maximum generated voltage. The maximum voltage is expected to be at the resonance of the beam (263 Hz) because the piezoelectric strip is attached to the HFB, so when the HFB reaches the resonance, the maximum deflection will occur, which means higher stresses and strains on the piezoelectric strip.

It is noticed that there are slight differences between the theoretical and experimental linear results. The reasons for such differences can be explained due to the deflection measurements. Theoretically, the tip magnets are assumed to be lumped with the cantilever beam’s mass, but experimentally, this is not the case exactly. Additionally, some of the differences could be from the lumped-parameter modeling in the theoretical analysis, which does not consider the rotations of the tip magnets, which slightly affect the experimental measurements. Another reason for such differences could be related to the difference in perfection between the theory and experimental implementation. In the theoretical model, we assumed both beams to be fully clamped-free, whereas in the experiment, such a boundary was not ideally clamped. However, the lumped-parameter modeling shows a strong capability to predict the response accurately.

#### 5.2.3. Nonlinear Analysis

**Figure 7:**
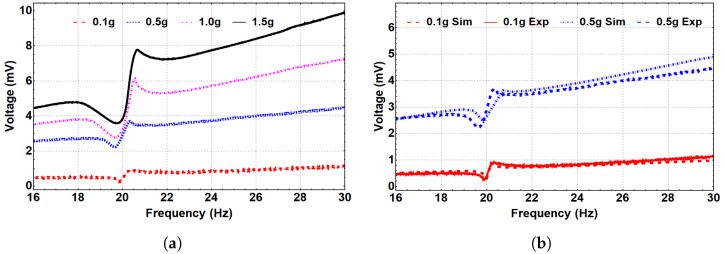
The frequency–voltage curve for different excitation levels at the monostable regime (d=35 mm). (**a**) Experimental results. (**b**) Simulated results with experimental validation.

The system’s dynamics were investigated at the three regimes: monostable, bistable, and threshold, with corresponding separation distances of 35 mm, 8 and 5 mm, and 15 mm, respectively. The generated frequency–voltage curves were extracted at the three regimes to show the frequency up-converter concept, where the voltage was generated at the LFB frequency range while the piezoelectric layer is attached to the HFB due to the magnetic coupling effect. At the monostable range, the separation distance was set to 35 mm, and the system was excited at different excitation levels, as shown in Figure 7a. Even though the piezoelectric strip was attached to the HFB, the voltage signal was generated at the LFB frequency range, proving the concept of the frequency up-conversion. Additionally, it is noted that higher output voltage was generated at higher excitation levels. Furthermore, nonlinear hardening behavior is shown with increasing the excitation level. Figure 7b shows that the simulated results agree with the experimental results at selected excitation levels of 0.1 and 0.5 g, which validates our theoretical model.

**Figure 8:**
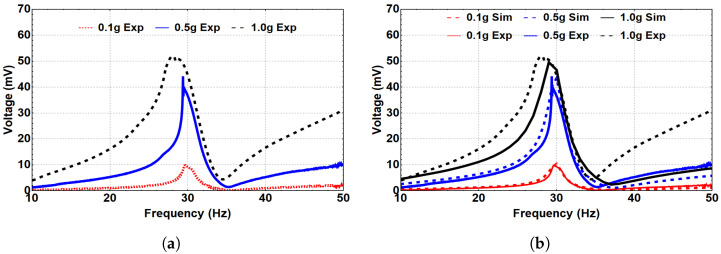
The frequency–voltage curve for different excitation levels at the bistable regime (d=8 mm). (**a**) Experimental results. (**b**) Simulated results with experimental validation.

Next, we investigated the system’s dynamic behavior in the bistable range. Toward this end, the system was subjected to different excitation levels at an 8 mm separation distance, as represented in Figure 8. As a result, a hardening behavior is shown where the natural frequency is shifted to reach 30 Hz at the 0.1 g excitation level, compared to 20.8 Hz for the linear harvester. This shift in the natural frequency occurs because of the change in the distance between the two magnets in the coefficient $\alpha_{1}$ of the magnetic force. With a further increase in the excitation level, the quadratic nonlinearity became dominant, and the natural frequency shifted to the left, indicating a softening behavior, as shown in Figure 8a. Moreover, the generated voltage in the bistable range significantly increased to reach 52 mV, compared to 6 mV for the monostable range at a 1.0 g excitation level. Figure 8b shows the simulated results and the experimental validation is in good agreement for various excitation levels.

**Figure 9:**
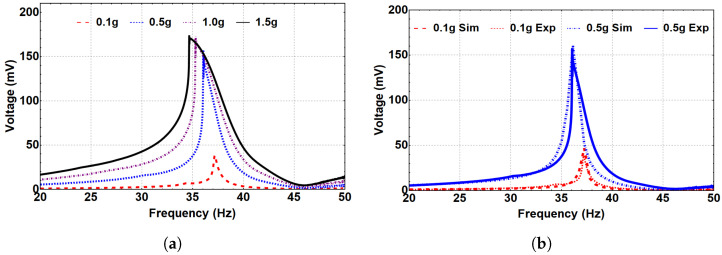
The frequency–voltage curve for different excitation levels at the bistable regime (d=5 mm). (**a**) Experimental results. (**b**) Simulated results with experimental validation.

Then, a separation distance of 5 mm was selected to investigate further the system’s dynamic behavior in the bistable regime. Therefore, the system was subjected to different excitation levels, as shown in Figure 9. At 0.1 g, the natural frequency increased to 37.2 Hz, as shown in Figure 9a, compared to 20.8 Hz for the linear harvester, indicating a hardening behavior. This increment was due to the change in the distance between the two magnets in the coefficient $\alpha_{1}$ of the magnetic force at low excitation levels, as reported previously. The natural frequency significantly increased at this separation distance compared to d=8 mm at the same excitation level. This could be related to the higher magnetic coupling between the two beams at a smaller separation distance. When the excitation level increased, a softening behavior was noticed, which was more significant than the previous case at d=8 mm. Additionally, the generated voltage significantly increased to around 170 mV at 1.5 g. The match between the experimental and simulated generated voltage at 0.1 and 0.5 g is presented in Figure 9b, and it shows good agreement that validates the theoretical model.

**Figure 10:**
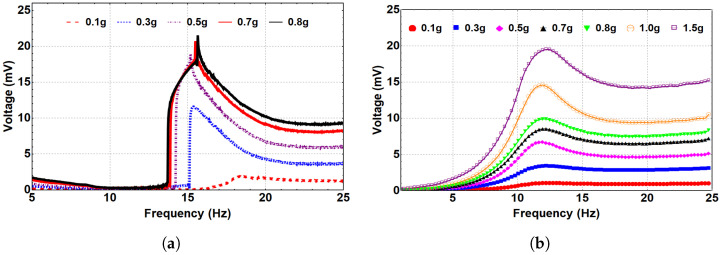
The frequency–voltage curves for different excitation levels at the threshold regime (d=15 mm). (**a**) Experimental results. (**b**) Simulated results.

Figure 10 shows the experimental and simulated generated voltage at the threshold regime where $d_{th}=15$ mm. The system response of this region was investigated at different excitation levels. At 0.1 g, the natural frequency dropped to 17 Hz, reflecting a softening behavior, compared to 20.8 Hz for the linear harvester because of the separation distance effect in the coefficient $\alpha_{1}$ of the magnetic force, so the natural frequency decreases, as shown in Figure 10a. Additionally, by increasing the excitation level, the natural frequency starts to increase after 0.5 g, so a nonlinear hardening behavior is shown, indicating an interesting nonlinear combined behavior of softening and hardening. The nonlinear combined softening and hardening behavior can be related to the exchange in the dominance of the quadratic nonlinearity at low excitation levels to the cubic nonlinearity at higher excitation levels. Figure 10b shows the simulated generated voltage at different excitation levels for the same separation distance. Qualitatively, the simulated results show that the natural frequency dropped to reach a lower value than the linear harvester indicating softening behavior, whereas increasing the excitation level shows a hardening behavior which is different from the behavior of the experimental results shown in Figure 10a. This difference is because the model does not accurately match the experimental results in capturing the natural frequency at the threshold, as shown in Figure 5a, where the experimental natural frequency at $d_{th}=15$ mm is approximately 17 Hz, whereas it is around 12 Hz for the simulated natural frequency. However, it is observed that the frequency–voltage curves show some mismatch between the simulation and the experiment, which seems to increase as the excitation level increases. This mismatch can be related to the high nonlinearity in the system at the threshold distance, where the system transfers between the monostable to the bistable range. Such behavior is not easily predicted accurately with the lumped-parameter model. However, these results are still considered qualitatively in good agreement. Furthermore, compared to the monostable region, the threshold plots show higher generated voltage for both simulation and experiment. At 0.1 g, the experimentally generated voltage was 1 mV in the monostable region, whereas it reached 2 mV at the threshold, which is 100% higher. Additionally, at the threshold with a 0.8 g excitation level, the experimentally measured voltage output peaked at almost 22 mV, compared to 8 mV at a higher excitation level of 1.5 g in the monostable region. This higher voltage generated at the threshold can be related to the higher coupling due to the lower separation distance than the monostable region.

**Figure 11:**
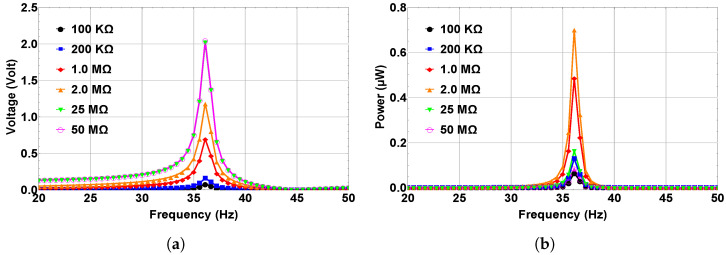
(**a**) Maximum output voltage. (**b**) Maximum output power at different resistance values for a separation distance of 5 mm and an excitation level of 0.5 g.

The effect of the external resistance variation on the maximum output voltage has been investigated. Figure 11a shows the change in the maximum output voltage while changing the external resistance of the system within the bistable range at d=5 mm at 0.5 g. The 200 kΩ was used in this study so far, and as shown in Figure 11a the maximum output voltage at this resistance value is approximately 0.16 V, and that was reported previously in Figure 9b. By decreasing the resistance to 100 kΩ, the produced voltage was reduced to roughly 0.09 V. When the resistance increased over 200 kΩ, the voltage also increased. At the resistance of 1 MΩ, the voltage went up to 0.7 V, then approximately doubled to reach 1.12 V at 2 MΩ. For the 25 MΩ, the produced voltage was 2.0 V. With a further resistance increase, the voltage no longer increased. Therefore, the maximum output voltage could be obtained at resistance of 25 MΩ. Moreover, the output power was calculated at different resistance values, as shown in Figure 11b. It is demonstrated that the power increases by increasing the resistance until it is maximized at R=2 MΩ with a value of approximately 0.76 μW, and any further increment in the resistance resulted in decreasing the output power, which dropped to approximately 0.185 μW and 0.15 μW for resistance levels of 25 and 50 MΩ, respectively. This observation concludes that 2 ΩM is the optimal resistance with maximum power. Therefore, it is worth mentioning that for maximizing the output power in practical applications, the optimum resistance that matches the internal impedance of the energy harvester needs to be investigated and used as the external resistance.

**Figure 12:**
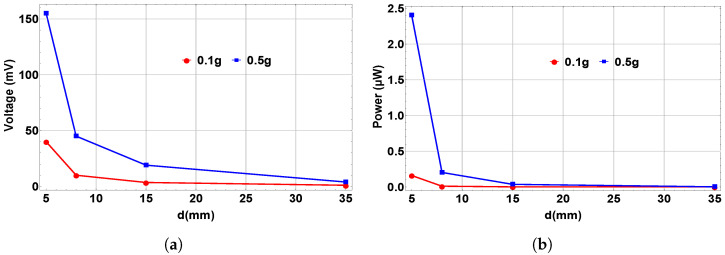
(**a**) The variation in the output voltage with separation distance at different excitation levels. (**b**) The variation in the output power with separation distance at different excitation levels.

In addition, the variations in output voltage and power with separation distance at different excitation levels are shown in Figure 12. We observed that both voltage and power increase when decreasing the distance between the two magnets from the monostable range toward the bistable range, and they are both maximized at the bistable region at d=5 mm. These results reflect the influences of magnet spacing on the output voltage and power. The magnetic force is magnified and the energy is maximized in the bistable range compared to the monostable and threshold ranges.

## 6. Conclusions

In conclusion, a frequency-up-converter piezoelectric energy harvester for low-vibration applications was investigated. The structure consists of two cantilever beams, a low-frequency beam (LFB), and a unimorph high-frequency beam (HFB) with an attached piezoelectric strip, and both beams are subjected to repulsive magnetic force from tip magnets. The structure was modeled as a two-degrees-of-freedom (2DOF) lumped-parameter model to study the static and dynamic behavior of the system. The static analysis resulted in a critical threshold separation distance of 15 mm that divides the system into monostable and bistable regions. The magnetic force transferred the vibrations and the harvested energy from a low-frequency range to a high-frequency range, which validated the concept of the frequency up-converter in energy harvesting applications. The linear response of the system without magnetic effect elevated natural frequencies of 20.8 Hz for the LFB and 263 Hz for the HFB. In the monostable region, a nonlinear hardening behavior was captured. In contrast, a combination of softening and hardening behaviors were reported at the threshold. In the bistable region, the softening behavior was dominant. The amount of voltage signal generated was maximized in the bistable region with a 1000% increment at d=8 mm and 4000% increment at d=5 mm compared to the monostable region at the 0.1g excitation level. Additionally, the generated power peaked at 2 MΩ, which indicated that this value represents the optimal resistance. Therefore, magnetic nonlinearity can be utilized for frequency up-converting to enhance the efficiency of low-frequency external-vibration energy harvesters. 

## Figures and Tables

**Table 1:** Physical and geometrical parameters of the frequency up-converter piezoelectric energy harvester.

Parameters	Symbol	Value
LFB (length × width × thickness)	$L_{1}\times b_{1}\times h_{1}$	(26×10×1) mm
LFB Young’s modulus	$E_{1}$	2.344 Gpa
LFB Density	$\rho_{1}$	1220 kg/m${}^{3}$
LFB Damping coefficient	$c_{1}$	0.0038 N s/m
HFB (length × width × thickness)	$L_{2}\times b_{2}\times h_{2}$	(19×10×1.6) mm
HFB Young’s modulus	$E_{2}$	69.0 Gpa
HFB Density	$\rho_{2}$	2700 kg/m${}^{3}$
HFB Damping coefficient	$c_{2}$	0.38 N s/m
Piezoelectric (length × width × thickness)	$L_{p}\times b_{p}\times h_{p}$	(19×7×0.02) mm
Piezoelectric Young’s modulus	$E_{p}$	2450 Mpa
Piezoelectric Density	$\rho_{p}$	1780 kg/m${}^{3}$
Resistance	*R*	200 kΩ
Magnets side length	$L_{m}$	8.0 mm
Magnetic moment	$q_{1}=q_{2}$	0.5 A${}^{2}$/m
Piezo Strain Constant	$d_{31}$	$23\times 10^{-12}$
Piezoelectric Laminate permittivity	$\epsilon_{33}$	$3250\times 8.854\times 10^{-12}$

## Data Availability

Data available on request from the authors.

## Appendix A. The Coefficients of the Expanded Magnetic Force

(A1)
$$\begin{array}{c} \begin{array}{cc} \alpha_{1} & =\frac{F_{R}\left(d^{2}-4Y_{s}^{2}\right)}{{\left(d^{2}+Y_{s}^{2}\right)}^{7/2}}\\ \alpha_{2} & =\frac{5F_{R}Y_{s}\left(-3d^{2}+4Y_{s}^{2}\right)}{2{\left(d^{2}+Y_{s}^{2}\right)}^{9/2}}\\ \alpha_{3} & =\frac{5F_{R}\left(d^{4}-12d^{2}Y_{s}^{2}+8Y_{s}^{4}\right)}{2{\left(d^{2}+Y_{s}^{2}\right)}^{11/2}}\\ \alpha_{4} & =\frac{35F_{R}Y_{s}\left(5d^{4}-20d^{2}Y_{s}^{2}+8Y_{s}^{4}\right)}{8{\left(d^{2}+Y_{s}^{2}\right)}^{13/2}}\\ \alpha_{5} & =\frac{7F_{R}\left(5d^{6}-120d^{4}Y_{s}^{2}+240d^{2}Y_{s}^{4}-64Y_{s}^{6}\right)}{8{\left(d^{2}+Y_{s}^{2}\right)}^{15/2}}\\ \alpha_{6} & =\frac{21F_{R}Y_{S}\left(-35d^{6}+280d^{4}Y_{s}^{2}-336d^{2}Y_{s}^{4}+64Y_{s}^{6}\right)}{16{\left(d^{2}+Y_{s}^{2}\right)}^{17/2}}\\ \alpha_{7} & =\frac{15F_{R}\left(7d^{8}-280d^{6}Y_{s}^{2}+1120d^{4}Y_{s}^{4}-896d^{2}Y_{s}^{6}+128Y_{s}^{8}\right)}{16{\left(d^{2}+Y_{s}^{2}\right)}^{19/2}}\\ \alpha_{8} & =\frac{165F_{R}Y_{s}\left(63d^{8}-840d^{6}Y_{s}^{2}+2016d^{4}Y_{s}^{4}-1152d^{2}Y_{s}^{6}+128Y_{s}^{8}\right)}{128{\left(d^{2}+Y_{s}^{2}\right)}^{21/2}}\\ \alpha_{9} & =\frac{55F_{R}\left(-21d^{10}+1260d^{8}Y_{s}^{2}-8400d^{6}Y_{s}^{4}+13440d^{4}Y_{s}^{6}-5760d^{2}Y_{s}^{8}+512Y_{s}^{10}\right)}{128{\left(d^{2}+Y_{s}^{2}\right)}^{23/2}} \end{array} \end{array}$$
